# Nocturnal enuresis as the initial symptom of life-threatening arrhythmia: a case report

**DOI:** 10.3399/bjgpopen18X101624

**Published:** 2018-12-12

**Authors:** Hiroshi Imamura, Hiroshi Kamijo, Kenichi Nitta, Ayako Okada

**Affiliations:** 1 Professor, Department of Emergency and Critical Care Medicine, Shinshu University School of Medicine, Matsumoto, Japan; 2 Doctor, Emergency and Critical Care Medicine, Shinshu University School of Medicine, Matsumoto, Japan; 3 Doctor, Emergency and Critical Care Medicine, Shinshu University School of Medicine, Matsumoto, Japan; 4 Doctor, Department of Cardiology, Shinshu University School of Medicine, Matsumoto, Japan

**Keywords:** nocturnal enuresis, ventricular fibrillation, Brugada syndrome, primary health care, general practice

## Introduction

Nocturnal enuresis affects 15–﻿20% of 5-year-old children, 5% of 10 year old children, and 1% to 2% of people aged ≥15 years. Nocturnal enuresis is rarely recognised as a serious event and is unusual in an adult. Brugada syndrome is a rare arrhythmia disease characterised by a specific electrocadiographic pattern and an increased risk of sudden cardiac arrest due to ventricular fibrillation in apparently healthy young adults^1^. Symptoms associated with Brugada syndrome include sudden cardiac death, palpitations, and chest discomfort. These symptoms often occur during rest or sleep. Here, the authors present what they believe is the first reported case of Brugada syndrome presenting as nocturnal enuresis in an adult.

## Case report

**Figure 1.**
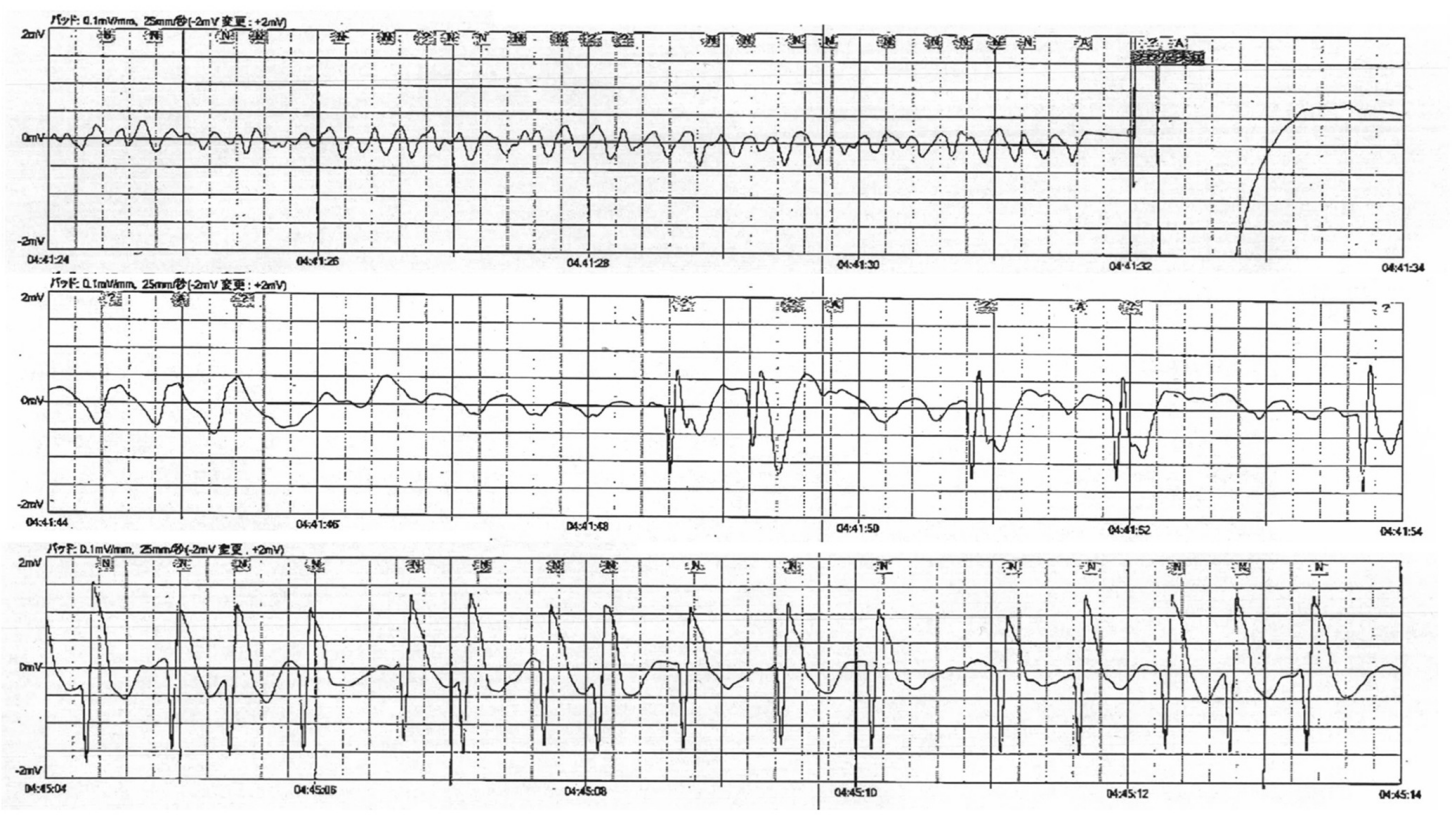
Electrocardiogram recorded by emergency responders showing ventricular fibrillation. After electrical shocks were delivered (the first column) and epinephrine was administered, spontaneous circulation was restored (the last column).

A 50 year old, previously healthy man was transferred to the emergency department of Shinshu University Hospital following resuscitation from ventricular fibrillation in September 2013. One morning in early summer of 2008, he woke up to find that he had experienced enuresis. Since then he experienced two to three additional episodes of nocturnal enuresis, which always occurred in the spring or summer. He did not consult with anyone about enuresis. At 0340 hours (h) on the day of admission, he awoke and found the bed was wet. He slept again after he changed his underwear. At 0416 h, his wife noticed that he suddenly groaned and became unresponsive. His wife immediately started basic life support. At 0436 h when emergency medical technicians arrived, the patient was found to be in ventricular fibrillation (Figure 1). At 0447 h, he was successfully resuscitated after two defibrillations and administration of epinephrine. At 0511 h, following hospital admission, he was unresponsive. Physical examination and laboratory test results were otherwise unremarkable. Coronary angiography showed normal coronary arteries, while an echocardiogram showed no structural heart disease and normal ventricular function. He underwent therapeutic hypothermia and was extubated on the fourth hospital day without neurological deficit.

The electrocardiogram showed sinus rhythm with transient coved-type ST elevation in the V2 lead, which is characteristic of Brugada syndrome type I.^2^ On a different day, his electrocardiogram showed saddleback type ST elevation. The patient did not have a history of syncope, seizures, chest discomfort, or nocturnal agonal respiration, nor was there a family history of sudden cardiac death. The diagnosis of Brugada syndrome was made. An implantable cardioverter defibrillator (ICD) was inserted.

When last followed up in July 2016, the patient was in good condition. His bed was dry even during an episode when an ICD shock was delivered to treat ventricular fibrillation during the night-time in May 2015.

## Discussion

Brugada syndrome is a rare inherited arrhythmia disease characterised by an increased risk of ventricular fibrillation in the absence of structural heart disease^1^. Clinical diagnosis is based on a specific electrocardiographic pattern consisting of coved-type ST-segment elevation, followed by a negative T wave in the right precordial leads. The clinical spectrum of Brugada syndrome patients ranges from asymptomatic to sudden cardiac death. Arrhythmia usually begins at an average age of 40 years, and sudden death typically occurs during sleep. Males are more commonly affected than females. Mutations involving cardiac sodium channels are identified in approximately 25% of cases. Symptoms associated with Brugada syndrome include ventricular fibrillation or aborted sudden cardiac death, nocturnal agonal respiration, palpitations, and chest discomfort.^3^ These symptoms often occur during rest or sleep, during a febrile state, or under vagotonic conditions, but rarely during exercise. It has been reported that there is a significant peak from spring to early summer in terms of the occurrence of ventricular fibrillation.^4^

The present patient had no symptoms other than nocturnal enuresis before sudden cardiac arrest. It appears that his nocturnal enuresis was the result of urinary incontinence caused by self-terminating ventricular fibrillation episodes during sleep. The seasonal pattern of his nocturnal enuresis was the same as that of ventricular fibrillation in patients with Brugada syndrome. He was, fortunately, resuscitated and had a good neurological recovery because of prompt bystander resuscitation and electrical shock. Berul *et al* ^5^ reported a 4-year-old male with nocturnal enuresis secondary to congenital complete heart block. Their patient experienced somnolence and difficulty awaking in the morning in addition to bedwetting. The authors speculated that bedwetting was secondary to excessive somnolence and difficulty with arousal due to low cardiac output. There has been no other reported case of arrhythmia in which the initial symptom was nocturnal enuresis. Nocturnal enuresis, to the authors' knowledge, has never been reported as the initial symptom of an adult case of arrhythmia. Nocturnal enuresis is rarely recognised as a serious event and is unusual in an adult. When an adult or even a pediatric patient is seen who complains of nocturnal enuresis, disorders that have bedwetting as a symptom (termed 'nocturnal incontinence') have to be excluded by a thorough history, examination, and urinalysis.

## Conclusion

This case highlights the need to be mindful that nocturnal enuresis can occur due to cardiovascular or neurogenic causes. Physicians should note that bedwetting in an adult may be the manifestation of a potentially fatal arrhythmic event while sleeping at night.
